# Genome-wide association study of paclitaxel and carboplatin disposition in women with epithelial ovarian cancer

**DOI:** 10.1038/s41598-018-19590-w

**Published:** 2018-01-24

**Authors:** Bo Gao, Yi Lu, Annemieke J. M. Nieuweboer, Hongmei Xu, Jonathan Beesley, Ingrid Boere, Anne-Joy M. de Graan, Peter de Bruijn, Howard Gurney, Catherine J. Kennedy, Yoke-Eng Chiew, Sharon E. Johnatty, Philip Beale, Michelle Harrison, Craig Luccarini, Don Conroy, Ron H. J. Mathijssen, Paul R. Harnett, Rosemary L. Balleine, Georgia Chenevix-Trench, Stuart Macgregor, Anna de Fazio

**Affiliations:** 1 Department of Gynaecological Oncology, Westmead Hospital, Sydney, Australia; 2 The Westmead Institute for Medical Research, Sydney Medical School, The University of Sydney, Sydney, Australia; 3 QIMR Berghofer Medical Research Institute, Brisbane, Australia; 4 Department of Medical Oncology, Erasmus MC Cancer Institute, Rotterdam, The Netherlands; 5 Unaffiliated, Boston, USA; 6 Crown Princess Mary Cancer Centre, Westmead Hospital, Sydney, Australia; 7 Chris O’Brien Lifehouse, Sydney, Australia; 8 Centre for Cancer Genetic Epidemiology, Department of Oncology, Cambridge University, Cambridge, UK; 9 Sydney West Translational Cancer Research Centre, Sydney, Australia; 10 Pathology West, Institute for Clinical Pathology and Medical Research (ICPMR), Westmead, Sydney, Australia

## Abstract

Identifying single nucleotide polymorphisms (SNPs) that influence chemotherapy disposition may help to personalize cancer treatment and limit toxicity. Genome-wide approaches are unbiased, compared with candidate gene studies, but usually require large cohorts. As most chemotherapy is given cyclically multiple blood sampling is required to adequately define drug disposition, limiting patient recruitment. We found that carboplatin and paclitaxel disposition are stable phenotypes in ovarian cancer patients and tested a genome-wide association study (GWAS) design to identify SNPs associated with chemotherapy disposition. We found highly significant SNPs in *ABCC2*, a known carboplatin transporter, associated with carboplatin clearance (asymptotic *P* = 5.2 × 10^6^, empirical *P* = 1.4 × 10^−5^), indicating biological plausibility. We also identified novel SNPs associated with paclitaxel disposition, including rs17130142 with genome-wide significance (asymptotic *P* = 2.0 × 10^−9^, empirical *P* = 1.3 × 10^−7^). Although requiring further validation, our work demonstrated that GWAS of chemotherapeutic drug disposition can be effective, even in relatively small cohorts, and can be adopted in drug development and treatment programs.

## Introduction

Large inter-individual variation in drug disposition has been reported for most chemotherapeutic drugs at standard dosing schedules^1^, including paclitaxel and carboplatin^2,3^. For women with advanced epithelial ovarian cancer (EOC), the standard of care is cytoreductive surgery and six cycles of paclitaxel and carboplatin chemotherapy^4,5^. Despite a high initial response rate (~75%), the median progression-free survival is between 16–21 months and there is a wide range in median overall survival reported, between 31–57 months^6^. There is also significant variability in chemotherapy side effects, some with a genetic component. For example, more haematological and neurological toxicities have been reported in Asian ovarian cancer patients compared to European patients^5,7,8^.

It may be hypothesized that variation in drug disposition is a contributing factor in variable treatment effects in EOC. There have been several well-studied associations between germline genetic variants and chemotherapeutic drug toxicity, leading to the inclusion of pharmacogenomic information in the US Food and Drug Administration (FDA) advice to prescribers for drugs including Irinotecan and Capecitabine^9–11^. In examples to date, these genetic variants were identified using a candidate gene approach, focusing on genes involved in drug metabolism. However, this approach requires *a priori* biological knowledge and will not reveal novel biomarkers or associations. The aim of this study was to identify novel germline genetic variants that influence carboplatin and paclitaxel disposition in ovarian cancer patients using a genome-wide association study (GWAS) approach.

## Results

### Patient selection and characteristics

The clinical characteristics of the two patient cohorts in this study, the Australian and Dutch cohorts, were similar, except that Dutch patients had higher body weight (due in part to increased height), resulting in higher paclitaxel doses (Table 1). The median creatinine level was lower (*P* = 0.002) in the Australian cohort (although both were within normal range), resulting in higher estimated glomerular filtration rate (GFR) (*P* = 0.003).

**Table 1:** Demographic and biological characteristics of patient cohorts.

	Normal	Median (range)		*P* ^a^
	range	Australian cohort	Dutch cohort	
Number of patients		61	35	
Ancestry				
European		43 (70.5%)	35 (100%)	
Asian		14 (23.0%)	0 (0.0%)	
Others		4 (6.6%)	0 (0.0%)	
Treatment				
Carboplatin + paclitaxel		55 (90.2%)	35 (100%)	
Carboplatin only		6 (9.8%)	0 (0.0%)	
Pharmacokinetic sampling				
Cycle 1		61 (100%)	N/A^b^	
Cycle 3^c^		7 (11.5%)		
Age		56 (19–74)	57 (38–74)	
Height (cm)		161 (148–174)	167 (150–179)	0.918
Weight (kg)		64 (36–120)	75 (53–111)	0.015
Total bilirubin (µmol/L)	≤20	6 (2–13)	6 (6–13)	0.430
Albumin (g/L)	35–50	40 (20–49)	N/A^d^	N/A
Alanine amino transaminase (U/L)	≤47	28 (6–159)	22 (7–55)	0.779
Aspartate amino transaminase (U/L)	≤45	40 (14–242)	24 (17–45)	0.016
Creatinine (µmol/L)	55–105	60 (42–165)	69 (45–118)	0.002
Glomerular filtration rate (mL/min)	90–120	80 (22–143)	70 (36–107)	0.003

^a^*P*-values were based on independent sample median test.

^b^Paclitaxel sampling was performed in different treatment cycles in the Dutch cohort.

^c^Seven patients had carboplatin and paclitaxel sampling performed during both cycle 1 and cycle 3.

^d^Albumin level results were available from only eight patients in the Dutch cohort.

### A significant inter-individual, but small intra-individual variability in paclitaxel and carboplatin disposition

**Figure 1:**
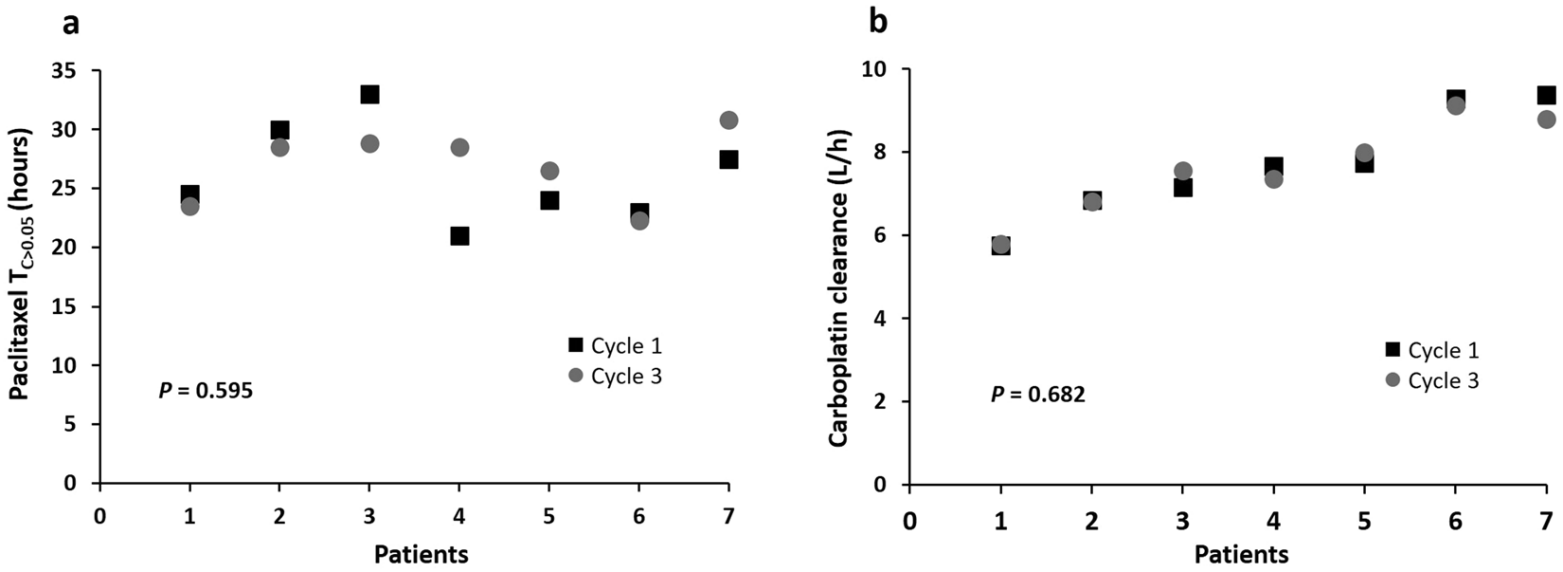
Pharmacokinetic parameter intra-individual variability. (**a**) Time of paclitaxel concentration >0.05 µmol/L (T_C>0.05_) and (**b**) carboplatin clearance from cycle one and three of chemotherapy in seven Australian patients, analysed by paired sample t test. The patients are ordered according to increasing carboplatin clearance rate.

The paclitaxel and carboplatin pharmacokinetic (PK) parameters were summarized in Tables 2 and 3, respectively. These results were consistent with published data^12,13^. Significant inter-individual variability was observed in paclitaxel T_C>0.05_ (Time of paclitaxel concentration > 0.05 µmol/L) (>2-fold) and carboplatin clearance (~5-fold). In contrast, there were no statistically significant differences of paclitaxel T_C>0.05_ (*P* = 0.595) (Fig. 1a) and carboplatin clearance (*P* = 0.682) (Fig. 1b) between two treatments in the seven Australian patients with PK sampling performed at both cycle one and three. These results demonstrated that chemotherapeutic drug disposition was a stable phenotype in individuals (at least over a six-week period), and supported the hypothesis that there is a genetic component underlying drug disposition.

**Table 2:** Paclitaxel pharmacokinetic parameters.

	**Median (range)**		***P - value*** ^**a**^
	**Australian cohort**	**Dutch cohort**	
Dose (mg)	290 (220–350)	320 (230–390)	<0.001
Infusion time (h)	3.2 (2.8–3.9)	3.4 (2.5–4.5)	0.224
Infusion rate (mg/h)	90 (71–110)	98 (63–135)	0.153
CL at the end of infusion (L/h)	9.9 (5.2–17.3)	11.2 (5.2–20.5)	0.209
T_C>0.05_ (h)	30.3 (21.0–47.3)	27.8 (20.5–46.0)	0.094
AUC_0-24_ (mg/L*h)	16.4 (10.5–26.1)	15.3 (11.3–27.1)	0.402
C_max_ (mg/L)	4.9 (3.2–7.5)	4.9 (3.0–8.9)	1.000

^a^*P*-values were based on the independent sample median test.

Abbreviations: CL, clearance; T_C>0.05_, time of paclitaxel concentration >0.05 µmol/L; AUC_0-24_, area under concentration-time curve of the first 24 hours; C_max_, maximum paclitaxel concentration.

**Table 3:** Carboplatin pharmacokinetic parameters.

Parameters	Median	Range
Dose (mg)	680	300–900
CL (L/h)	7.5	2.8–12.9
V (L)	15.5	4.0–32.0
C_max_ (mg/L)	38.0	18.6–57.1
AUC_0–24_ (mg/L*h)	89.9	60.1–129.4

Abbreviations: CL, clearance; V, volume of distribution; C_max_, maximum concentration; AUC_0-24_, area under concentration-time curve of the first 24 hours.

### Correlations between drug disposition and hematological toxicity

**Figure 2:**
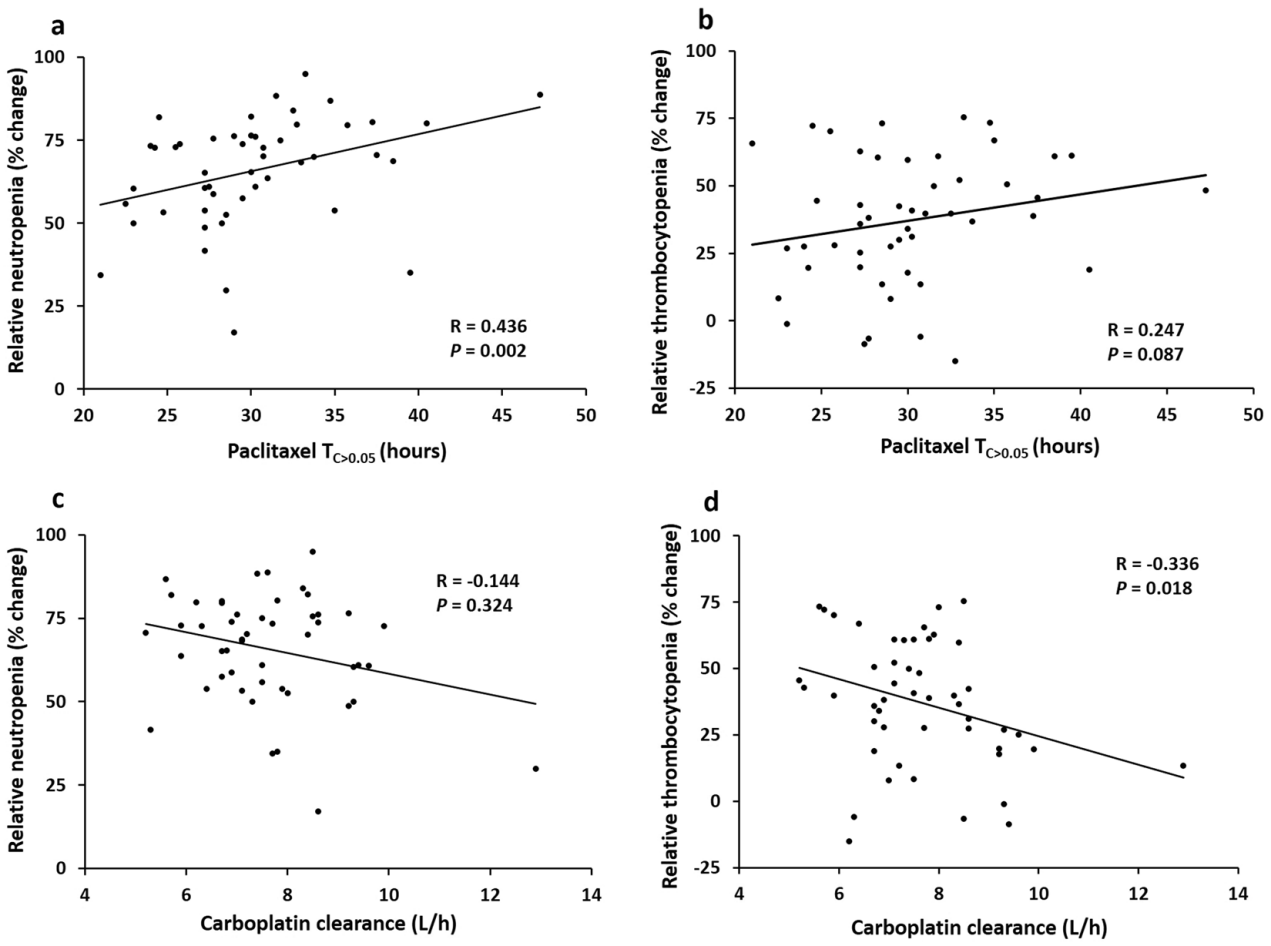
Associations between paclitaxel and carboplatin disposition and haematological toxicities. (**a**) Association between time of paclitaxel concentration >0.05 µmol/L (T_C>0.05_) and relative neutropenia; (**b**) association between T_C_ > 0.05 and relative thrombocytopenia; (**c**) association between carboplatin clearance and relative neutropenia and (**d**) association between carboplatin clearance and relative thrombocytopenia, analysed by simple linear regression analysis in 49 Australian patients receiving paclitaxel and carboplatin combination chemotherapy.

Hematological assessment after the first cycle of chemotherapy was performed in 49 Australian patients receiving combination chemotherapy. Paclitaxel T_C>0.05_ was associated with relative neutropenia (Fig. 2a) (R = 0.436, *P* = 0.002) but not with relative thrombocytopenia (Fig. 2b) (R = 0.247, *P* = 0.087). On the other hand, the correlation between carboplatin clearance and relative neutropenia was not statistically significant (Fig. 2c) (R = −0.144, *P* = 0.324), but there was a statistically significant inverse correlation between carboplatin clearance and relative thrombocytopenia (Fig. 2d) (R = −0.336, *P* = 0.018) by simple linear regression analysis. These findings are in accordance with previous publications^14–16^ and confirmed paclitaxel T_C>0.05_ is a good predictive marker for severe neutropenia, whereas carboplatin clearance predicts thrombocytopenia, using the population pharmacokinetic modelling we applied.

### GWAS identified novel polymorphisms associated with paclitaxel exposure

We found that ~27% variability in paclitaxel T_C>0.05_ was accounted for by paclitaxel dose, GFR and AST (aspartate amino transaminase), therefore tested each SNP for its association with paclitaxel T_C>0.05_ without and with adjustment for these three covariates. Thirty-seven patients of European ethnicity and 14 of Asian ethnicity from the Australian cohort, and 35 patients of European ethnicity from the Dutch cohort were available for analysis. Six Dutch patients were missing AST values therefore 29 were included in the adjusted analysis.

**Figure 3:**
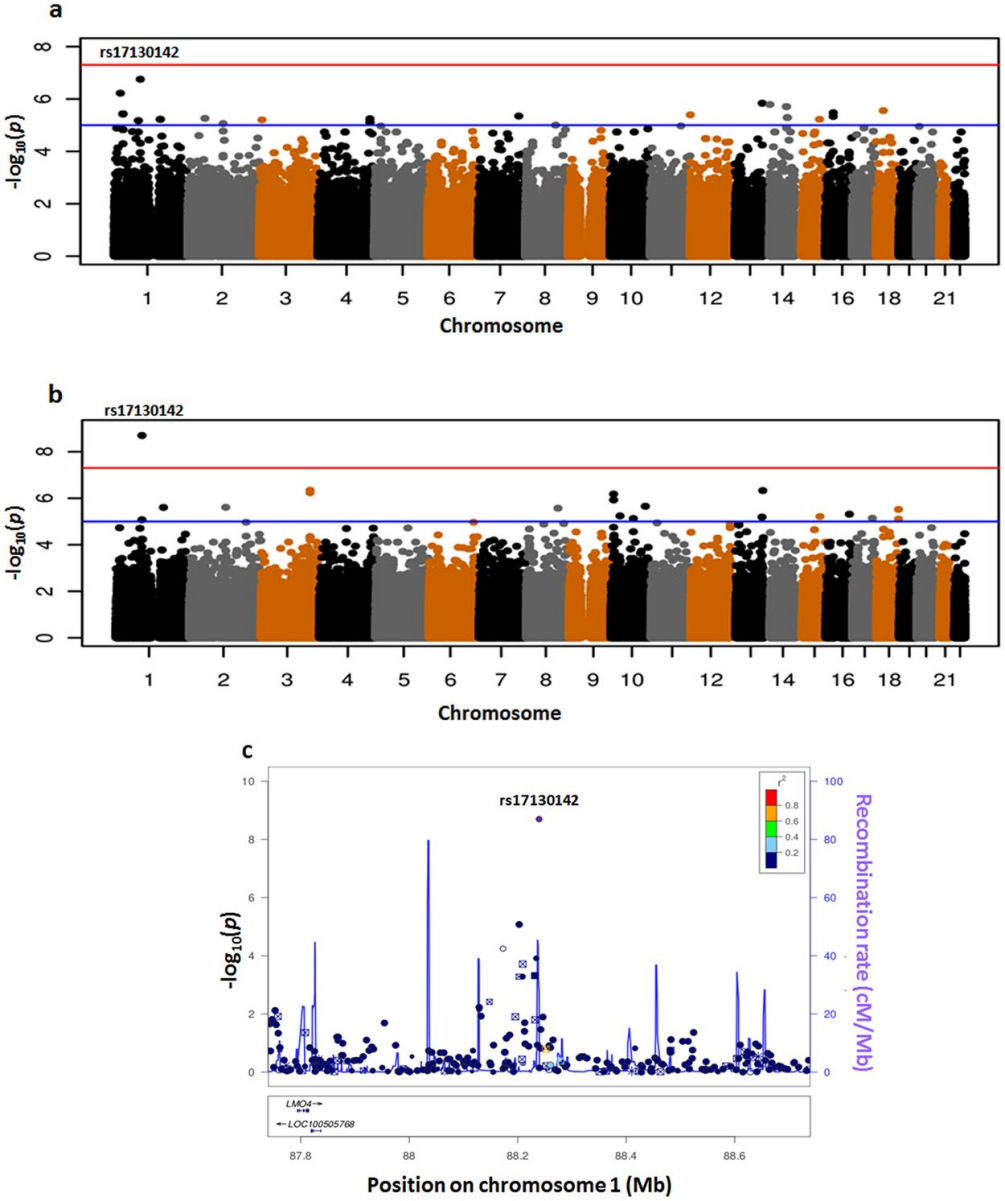
Paclitaxel GWAS. Manhattan plots of paclitaxel disposition GWAS (**a**) unadjusted and (**b**) adjusted for paclitaxel dose, glomerular filtration rate (GFR) and aspartate amino transaminase (AST); (**c**) LocusZoom plot for rs17130142 from the adjusted paclitaxel GWAS. The color shading indicates the strength of LD (r^2^) from the reference of 1000 Genome Project (CEU samples) between the corresponding SNP and the top SNP, rs17130142. The red horizontal line indicates the genome-wide significance threshold of *P* = 5 × 10^−8^; the blue line is the threshold for putative association (*P* = 10^−5^).

Manhattan plots, showing SNP association *P-*values from the meta-analysis (-log_10_(*p*)) against genomic position, in unadjusted and adjusted analyses are presented in Fig. 3a and b. The genomic control parameters were 1.01 and 1.02 respectively, suggesting that there was little population substructure or other artefacts. In the unadjusted GWAS (Fig. 3a), 25 SNPs were found to be associated with paclitaxel T_C>0.05_ with *P* < 1 × 10^−5^ (Supplementary Table S1). The SNP with the highest statistical significance was rs17130142. The *P* value from meta-analysis was 1.8 × 10^−7^. We ran 10^7^ permutations to correct for the asymptotically approximated results and empirical p-value remained unchanged. In the adjusted analysis (Fig. 3b), 19 SNPs were associated with paclitaxel T_C>0.05_ with *P* < 1 × 10^−5^ (Supplementary Table S2). Rs17130142 remained the most statistically significant polymorphism.

Rs17130142 is an intergenic polymorphism on chromosome 1 (Fig. 3c). It has a minor allele frequency (MAF) ~5% in European patients (7% and 3% in the Australian and Dutch cohort respectively), but was monomorphic in the 14 Asian patients. The minor allele A was associated with increased paclitaxel T_C>0.05_ in European patients from both the Australian cohort (effect = 8.58, *P* = 6.5 × 10^−6^) and the Dutch cohort (effect = 18.24, *P* = 7.7 × 10^−5^). In the meta-analysis, it met the criteria for genome-wide statistical significance (*P* = 2.0 × 10^−9^). Due to the small number of heterozygous patients (5 from 37 Australian patients and 2 from 29 Rotterdam patients), the empirical p-value after 10^7^ permutations dropped to 1.3 × 10^−7^.

### *ABCC2* polymorphisms associated with carboplatin clearance

Consistent with published data^17^, we found GFR accounted for >90% variability in carboplatin clearance. Therefore, we tested association between each SNP and carboplatin clearance unadjusted and adjusted for GFR. 42 European patients (1 removed due to low genotyping call rate) and 14 Asian patients in the Australian cohort were included in meta-analysis weighted by sample size.

**Figure 4:**
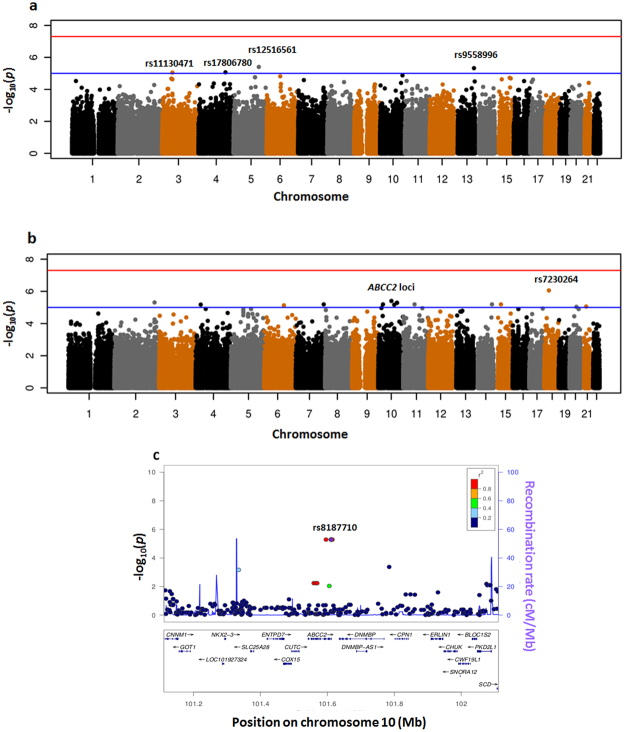
Carboplatin GWAS. Manhattan plots of carboplatin clearance GWAS (**a**) unadjusted and (**b**) adjusted for glomerular filtration rate; (**c**) LocusZoom plot for *ABCC2* loci from adjusted carboplatin GWAS. The colour shading indicates the strength of LD (r^2^) from the reference of 1000 Genome Project (CEU samples) between the corresponding SNP and the labelled SNP, rs8187710. The red horizontal line indicates the genome-wide significance threshold of *P* = 5 × 10^−8^; the blue line is the threshold for putative association (*P* = 10^−5^).

The genomic control parameters for the unadjusted and adjusted analysis were 1.00 and 1.03 respectively. In the unadjusted GWAS (Fig. 4a), four SNPs were found to be associated with carboplatin clearance with *P* < 1 × 10^−5^ (Supplementary Table S3). After adjusting for GFR, these associations were not statistically significant (*P* > 0.05), suggesting the putative associations found between these SNPs and carboplatin clearance were due to associations with GFR.

However, in the adjusted GWAS (Fig. 4b), 17 SNPs were found to be associated with carboplatin clearance with *P* < 1 × 10^−5^ (Supplementary Table S4). Of highest statistical significance was rs7230264 (*P* = 8.7 × 10^7^), a SNP that lies within an intron of RP11-739N10, a long intergenic non-coding RNA (lincRNA) of unknown function on chromosome 18. The minor allele A was associated with decreased carboplatin clearance and has a frequency of 12% in European patients and 25% in Asian patients.

Interestingly, a cluster of SNPs in complete linkage disequilibrium (r^2^ = 1) around *ABCC2* were found to be putatively associated with carboplatin clearance in 42 patients with European ancestry (*P* = 5.2 × 10^−6^) (Fig. 4c). They are monomorphic in 14 patients with Asian ancestry (Supplementary Table S4). Located on chromosome 10q24, *ABCC2* encodes a carboplatin efflux transporter ABCC2 (also known as multiple resistant protein 2 - MRP2) (see the locusZoom plot^18^ in Fig. 4c). Due to the small number of heterozygous patients for these SNPs (N = 3), we also ran 10^7^ permutations to correct for the asymptotically approximated results. The empirical *p* value from the permutation was 1.4 × 10^−5^. The details of the four SNPs in *ABCC2* are summarised in Table 4. Patients carrying the variant alleles at these SNPs had decreased carboplatin clearance (effect = −1.21; Supplementary Table S4), potentially leading to a higher carboplatin systemic exposure.

**Table 4:** Genetic polymorphisms in *ABCC2* associated with carboplatin clearance in the adjusted carboplatin GWAS.

dbSNP ID	Location	Transcript position	Coding position	Allele	Codons	Amino acid position	Amino acid
rs17222723	exon 25	3702	3563	T → A	GTG → GAG	1188	Val → Glu
rs8187707	exon 31	4627	4488	C → T	CAC → CAT	1496	His = His
rs8187710	exon 32	4683	4544	G → A	TGC → TAC	1515	Cys → Tyr
rs11816708	intergenic	N/A	N/A	T → C	N/A	N/A	N/A

Abbreviations: Val, valine; Glu, glutamate; His, histidine; Cys, cysteine; Tyr, tyrosine

## Discussion

Inherited genetic variation has an impact on drug absorption, metabolism and excretion, and therefore on pharmacodynamics. Identifying SNPs that influence chemotherapy disposition may help to personalize individual treatment. While GWAS has the advantage of being unbiased, they usually require a large sample size. Since most cytotoxic drugs have a short half-life and are given cyclically, multiple blood samples are required to adequately define drug disposition and systemic exposure. This tends to limit patient sample sizes in pharmacokinetic studies. Having demonstrated that carboplatin and paclitaxel disposition is a stable phenotype within an individual, we performed two separate GWAS of paclitaxel and carboplatin disposition and identified novel inherited genetic variants associated with drug disposition in ovarian cancer patients.

In the paclitaxel GWAS, we identified a novel polymorphism associated with paclitaxel T_C>0.05_ with genome-wide significance. Rs17130142 is located on chromosome 1 between *LOC100505768* (distance = 402 kb) and *PKN2* (Protein kinase N2) (distance = 910 kb). PKN2 encodes a transmembrane serine/threonine protein kinase. It plays a role in cell cycle progression and cytokinesis^19^. Being an intergenic polymorphism, the function and effects of rs17130142 are unknown, but it may regulate upstream or downstream genes and affect gene splicing, transcription factor binding, messenger RNA degradation, or the sequence of non-coding RNA^20,21^. Further fine mapping and functional analysis to determine the target gene and underlying mechanism is warranted.

In the adjusted carboplatin GWAS, four *ABCC2* SNPs in complete linkage disequilibrium were associated with carboplatin clearance. ABCC2 is localized in the apical luminal membrane of polarized epithelial cells of several excretory organs including liver, intestine and kidney^22^. It has an important function in the biliary excretion of endogenous metabolites. ABCC2 transports drug conjugated to glutathione including platinum agents^23^ and also exports many unconjugated substances^24^. *ABCC2* polymorphism has been found associated with response and survival in patients with gastric^25^, oesophageal^26^, ovarian^27^, non-small cell cancer lung cancer^28,29^. Among the four SNPs, rs8187710 (4544 G > A) in exon 32 is non-synonymous leading to a cysteine to tyrosine transition at position 1515 in the last cytoplasmic loop of ABCC2. In stably transfected human embryonic kidney cells (HEK293), a 4544 A clone showed higher accumulation of lopinavir, calcein and carboxyfluorescein diacetate (ABCC2 substrates) as well as reduced ABCC2 mediated efflux of lopinavir compared with a 4544G clone^30^. Increased risks of acute anthracycline-induced cardiac toxicity^31^ and congestive heart failure^32^ have also been reported in patients carrying the minor allele at rs8087710, who received doxorubicin chemotherapy, another ABCC2 substrate. As there is no physiological evidence of ABCC2 expression in the heart, increased cardiac toxicity could be caused by a reduced biliary elimination of doxorubicin, which normally accounts for 50% of its disposition^33^. Further supporting our findings, the major G allele of rs8187710 has been reported to be associated with inferior overall survival in 188 platinum-treated stage IV non-small-cell lung cancer (ASCO annual meeting 2012, abstract number 7586), presumably due to increased platinum clearance. These reports are in agreement with our finding that minor alleles of these *ABCC2* SNPs are associated with reduced ABCC2 transporter function, leading to decreased carboplatin clearance. This could be clinically relevant for platinum toxicity and response. Although fine mapping and functional analyses have not been performed, ABCC2 is an obvious candidate and it is reasonable to assume that it is the functionally relevant target of this GWAS finding.

Additional SNPs were found to be associated with carboplatin clearance in the adjusted GWAS. Among the SNPs listed in Supplementary Table S4, rs7230264 located in lincRNA RP11-739N10 on chromosome 18 was of the highest statistical significance. LincRNAs have been implicated in a wide range of cellular processes including gene transcription regulation^34^, post-transcriptional regulation^35^ and epigenetic regulation^36^. The role of lincRNA RP11-739N10 in relation to carboplatin clearance remains to be determined.

GWAS have been used to identify common DNA sequence variants that are associated with susceptibility to over 250 complex disease traits, using very large sample size of thousands to hundreds of thousands of patients. With few exceptions, the odds ratios (ORs) for the effects are typically in the range of 1.05 to 1.15. In contrast, GWAS studies of drug efficacy or safety with small patient cohorts, with some ranging from only 20 to 80 patients^37–40^, have revealed a substantial number of unanticipated and often striking signals. The large effects identified in pharmacogenomics studies are likely due to the lack of an evolutionary response to modern exogenous agents. Unlike genes associated with common disease, which are located on haplotype blocks that have been selected to overcome environmental influences and selection pressure over thousands if not millions of years, the genome has had very little time to adapt to exposure to prescription medications^41^. This could explain our significant findings in a relatively small patient cohort.

In conclusion, our GWAS approach identified SNPs putatively associated with carboplatin and paclitaxel disposition. Examination of the functional characteristics of the SNPs may provide novel pharmacogenomic biomarkers for personalized ovarian cancer treatment. More importantly, our findings support that GWAS of chemotherapeutic drug disposition can be effective even when performed in a relatively small number of samples and can be used to improve the drug candidate pipeline and boost the efficacy while limiting the toxicity of medications already in use. As genotyping and sequencing costs continue to decline, every effort should be made to incorporate comprehensive, unbiased pharmacogenomic analyses in strategies to optimise treatment outcome.

## Materials and Methods

### Patient population

Patients with EOC receiving paclitaxel and carboplatin (N = 55) or single agent carboplatin (N = 6), were recruited between 2009 and 2012 from Westmead Hospital and Royal Prince Alfred Hospital, Sydney, Australia. Key inclusion criteria were histologically or cytologically confirmed EOC; ECOG ≤ 2; adequate organ function and no concurrent use of moderate or strong cytochrome P450 and ATP-binding cassette transporter (ABC transporter) inhibitors or inducers. An independent paclitaxel pharmacokinetic (PK) cohort of 35 EOC patients was recruited through the Erasmus MC - Cancer Institute, Rotterdam, the Netherlands. This study was approved by the Medical Ethics Review Board Erasmus MC in the Netherlands and the Sydney West Area Health Service Human Research Ethics Committee (now known as the Western Sydney Local Health District Human Research Ethics Committee) in Australia. All experiments were performed in accordance with relevant guideline and regulations. Written informed consent was obtained from each patient.

Cremophor EL formulated paclitaxel (175 mg/m^2^) was administered intravenously (i.v.) over a planned 3-hour infusion. This was immediately followed by a 1-h infusion of carboplatin (AUC 5-6 mg/mL*min) in patients receiving combination chemotherapy. The carboplatin doses were calculated according to the method described by Calvert^42^ and glomerular filtration rate (GFR) was estimated using the Cockcroft-Gault formula^43^.

### Pharmacokinetic blood sampling and bioanalysis

Blood samples for PK analyses were taken in 10-mL ETDA tubes during the cycle one treatment for all patients. In consenting participants from the Australian cohort, additional PK blood samples were also collected during cycle three of chemotherapy (N = 7). In the Australian cohort, blood sampling was taken immediately before the start, 1.5 hours after the start, at the end of paclitaxel infusion, at the end of carboplatin infusion, ~2–3 hours after the end of carboplatin infusion and ~ 21 hours after the end of paclitaxel infusion respectively, with the precise time for each blood sampling recorded. In the Dutch cohort, blood sampling was performed before the start, 1.5 hours after the start, at the end and ~2–3 hours after the end of paclitaxel infusion.

Whole blood was centrifuged immediately for 10 minutes at 3,000 rpm (1,500 *g*) at 4 °C. For paclitaxel PK analysis, plasma was collected and stored at -80 °C. For carboplatin PK analysis, plasma was immediately transferred and centrifuged through a 30-kDa cut-off Amicon® ultrafiltrate filter (Millipore, Billerica, Massachusetts USA) for 15 min at 3,000 rpm at 20 °C. The preparation of ultrafiltrate was done immediately after blood collection to prevent the decrease of free carboplatin level due to *ex vivo* binding of carboplatin to plasma proteins and erythrocytes. All specimens were stored in cryovials at -80 °C until analysis.

Paclitaxel concentrations were measured using liquid chromatography - tandem mass spectrometry (LC-MS/MS) (Waters Chromatography B.V, Etten-Leur, the Netherlands) at the Laboratory of Translational Pharmacology, Department of Medical Oncology, Erasmus MC Cancer Institute, Rotterdam, the Netherlands^44^. Carboplatin levels were measured using a Varian 820 inductively coupled plasma mass spectrometry (ICP-MS) connected to a Varian SPS 3 auto-sampler as previously described with minor modification^45^ at Trace Inorganics Laboratory, Division of Analytical Laboratories, Sydney West Area Health Service, Australia.

### Population pharmacokinetic modelling

The paclitaxel and carboplatin concentration-time data were analysed using the non-linear, mixed-effects modelling program (NONMEM) version VI (ICON, Hanover, MD, USA). R (version 2.13 or above, R programming) and PsN (Perl Speaks for NONMEM, Uppsala) were used for data preparation, graphical analysis, model diagnostics. The first-order conditional estimation with interaction (FOCEI) method in NONMEM was employed for model runs.

A stepwise forward selection and backward elimination approach was used for both paclitaxel and carboplatin modelling. Generally, a base model without any covariates was developed. Random effects were treated as lognormal in distribution. Residual error was modelled as additive error, proportional error or a combination of these two. One, two or three compartment models were tested when necessary. After a best base model was found, covariate modeling was then performed. The covariates examined in the model included age, weight, GFR, albumin, bilirubin, alanine aminotransferase (ALT), aspartate aminotransferase (AST), haemoglobin, white blood cell (WBC) count, absolute neutrophil count (ANC) and platelet. Final model was assessed by a visual predictive check, comparing observed concentration *vs*. time data and the 90% confidence interval generated using 500 simulated concentration–time data sets.

One compartment model with proportional and additive residual error described carboplatin concentration data very well. The final model for paclitaxel is a 3-compartment model with proportional residual error^46^. Paclitaxel clearance is non-linear and it is not clear which time points are the most indicative of clinically important paclitaxel clearance. Time of paclitaxel concentration >0.05 µmol/L (T_C>0.05_) has been reported to be associated with both haematological toxicity and chemotherapy response and survival^14^, simulation was therefore performed to obtain T_C>0.05_ by introducing more frequent sampling schedules in the dataset.

### Haematological toxicity assessment

Baseline full blood counts (FBCs) were measured within 14 days prior to cycle one chemotherapy. Nadir FBCs were measured on day 14 ± 3 after cycle one chemotherapy. Haematological toxicities were assessed by relative neutropenia and thrombocytopenia, defined as the percentage reduction of nadir absolute neutrophil counts and platelet counts compared with baseline.

### DNA extraction and genotyping

DNA was extracted from whole blood using DNA Mini Kit (Qiagen, GmbH, Hilden, Germany). A total of 719,665 SNPs was genotyped on the Australian and Dutch samples using Illumina Human OmniExpress arrays (Illumina Inc, San Diego, CA USA). The following quality control steps were applied: (1) removing samples with >5% missing genotypes; (2) excluding any SNP with less than 1% MAF; (3) excluding any SNP that failed the Hardy-Weinberg Equilibrium (HWE) test at the significance level of *P* = 1 × 10^–7^; (4) excluding the SNPs with MAF > 5% when the per SNP no-call rate >5%, and SNPs with MAF < 5% when the per SNP no-call rate >1%. One Australian patient was removed from analysis due to low genotyping rate (~75%). A total of 610 255 autosomal SNPs were retained after quality control. In addition, we checked samples for cryptic relatedness using the software PLINK^47^ and genetic ancestry using EIGENSTRAT^48^. We confirmed that self-reported ethnicities were concordant with genetic ancestries for all samples.

### Statistical analysis

Linear regression was used to test the association between each SNP and the trait of interest, both adjusted and unadjusted for corresponding covariates, using PLINK^47^. Because meta-analysis has been shown to be more efficient than replication-based analysis^49^, we performed separate analysis within each population and then ran meta-analysis combining results from different populations weighted by sample sizes using METAL^50^. *P* values < 1 × 10^−5^ were considered as putative associations, and *P* values < 5 × 10^−8^ were regarded as genome-wide significance.

## Electronic supplementary material

Supplemetary data
