# Supplementary material for: Genome-wide association study of paclitaxel and carboplatin disposition in women with epithelial ovarian cancer
Source: Sci Rep. 2018 Jan 24;8:1508. doi: 10.1038/s41598-018-19590-w (PMC5784122; doi:10.1038/s41598-018-19590-w)
Supplement: Supplementary file 1 — Supplemetary data [file 41598_2018_19590_MOESM1_ESM.doc]

**Genome-wide association study of paclitaxel and carboplatin disposition in women with epithelial ovarian cancer**

Bo Gao^1,2,+^, Yi Lu^3,+^, Annemieke J.M. Nieuweboer^4^, Hongmei Xu^5^, Jonathan Beesley^3^, Ingrid Boere^4^, Anne-Joy M. de Graan^4^, Peter de Bruijn^4^, Howard Gurney^6^, Catherine J. Kennedy^1,2^, Yoke-Eng Chiew^1,2^, Sharon E. Johnatty^3^, Philip Beale^7^, Michelle Harrison^7^, Craig Luccarini^8^, Don Conroy^8^, Ron H. J. Mathijssen^4^, Paul R. Harnett^2,6,9^, Rosemary L. Balleine^2,9,10^, Georgia Chenevix-Trench^3,#^, Stuart Macgregor^3,#^ and Anna de Fazio^1,2,6,9,#^

^1^Department of Gynaecological Oncology, Westmead Hospital, Sydney, Australia

^2^The Westmead Institute for Medical Research, Sydney Medical School, The University of Sydney, Sydney, Australia

^3^QIMR Berghofer Medical Research Institute, Brisbane, Australia

^4^Department of Medical Oncology, Erasmus MC Cancer Institute, Rotterdam, the Netherlands

^5^Unaffiliated, Boston, USA

^6^Crown Princess Mary Cancer Centre, Westmead Hospital, Sydney, Australia

^7^Chris O’Brien Lifehouse, Sydney, Australia,

^8^Centre for Cancer Genetic Epidemiology, Department of Oncology, Cambridge University, Cambridge, UK

^9^Sydney West Translational Cancer Research Centre, Sydney, Australia

^10^Pathology West, Institute for Clinical Pathology and Medical Research (ICPMR), Westmead, Sydney, Australia

^+^ Bo Gao and Yi Lu contributed equally to this work

^#^ Georgia Chenevix-Trench, Stuart Macgregor and Anna de Fazio jointly supervised this work

| **Supplementary Table S1a.** SNPs with meta-analysis *P* value less than 1 × 10^-5^ in the unadjusted GWAS of paclitaxel disposition, stratified by cohorts. | | | | | | | | | | | | | | | |  |
| --- | --- | --- | --- | --- | --- | --- | --- | --- | --- | --- | --- | --- | --- | --- | --- | --- |
|  |  |  | **Australian cohort European** | | |  | **Dutch cohort European** | | | |  | **Australian cohort Asian** | | | | |
| **SNP** | **CHR** | **Weight** | **MAF** | **BETA** | ***P*** |  | **Weight** | **MAF** | **BETA** | ***P*** |  | **Weight** | **MAF** | **BETA** | ***P*** | |
| rs17130142 | 1 | 37 | 0.07 | 8.641 | 1.4×10^-4^ |  | 35 | 0.03 | 11.41 | 3.4×10^-4^ |  | NA | NA | NA | NA | |
| rs1614627 | 1 | NA | NA | NA | NA |  | 33 | 0.09 | 7.424 | 2.3×10^-6^ |  | 11 | 0.09 | 9.07 | 7.2×10^-2^ | |
| rs11840576 | 13 | 37 | 0.04 | 8.684 | 3.4×10^-3^ |  | 35 | 0.03 | 12.2 | 9.7×10^-5^ |  | NA | NA | NA | NA | |
| rs17211649 | 14 | NA | NA | NA | NA |  | 35 | 0.03 | 14.22 | 1.6×10^-6^ |  | NA | NA | NA | NA | |
| rs2056859 | 14 | NA | NA | NA | NA |  | 35 | 0.03 | 11.03 | 5.8×10^-4^ |  | 14 | 0.04 | 17.65 | 5.4×10^-4^ | |
| rs4899628 | 14 | NA | NA | NA | NA |  | 35 | 0.03 | 11.03 | 5.8×10^-4^ |  | 14 | 0.04 | 17.65 | 5.4×10^-4^ | |
| rs8083427 | 18 | 37 | 0.08 | 5.467 | 1.4×10^-2^ |  | 35 | 0.01 | 18.28 | 1.8×10^-5^ |  | 14 | 0.11 | 3.46 | 3.9×10^-1^ | |
| rs9938946 | 16 | 37 | 0.18 | 3.774 | 1.5×10^-2^ |  | 35 | 0.16 | 3.846 | 9.3×10^-3^ |  | 14 | 0.04 | 17.65 | 5.4×10^-4^ | |
| rs12134081 | 1 | 37 | 0.18 | 5.346 | 1.4×10^-3^ |  | 35 | 0.16 | 3.751 | 1.1×10^-2^ |  | 14 | 0.29 | 5.49 | 2.2×10^-2^ | |
| rs16838233 | 1 | 37 | 0.18 | 5.346 | 1.4×10^-3^ |  | 35 | 0.16 | 3.751 | 1.1×10^-2^ |  | 14 | 0.29 | 5.49 | 2.2×10^-2^ | |
| rs16838255 | 1 | 37 | 0.18 | 5.346 | 1.4×10^-3^ |  | 35 | 0.16 | 3.751 | 1.1×10^-2^ |  | 14 | 0.29 | 5.49 | 2.2×10^-2^ | |
| rs12425791 | 12 | 37 | 0.23 | 2.026 | 1.0×10^-1^ |  | 35 | 0.16 | 4.756 | 2.0×10^-4^ |  | 14 | 0.29 | 5.56 | 4.0×10^-3^ | |
| rs17169015 | 7 | NA | NA | NA | NA |  | 35 | 0.01 | 18.28 | 1.8×10^-5^ |  | 14 | 0.07 | 8.03 | 7.1×10^-2^ | |
| rs13331813 | 16 | 37 | 0.14 | 3.844 | 2.0×10^-2^ |  | 35 | 0.16 | 3.846 | 9.3 × 10^-3^ |  | 14 | 0.04 | 17.65 | 5.4×10^-4^ | |
| rs12879016 | 14 | 37 | 0.22 | 4.956 | 6.1×10^-4^ |  | 35 | 0.16 | 6.098 | 9.8×10^-5^ |  | 14 | 0.32 | -0.99 | 6.7×10^-1^ | |
| rs2205226 | 14 | 37 | 0.22 | 4.956 | 6.1×10^-4^ |  | 35 | 0.16 | 6.098 | 9.8×10^-5^ |  | 14 | 0.32 | -0.99 | 6.7×10^-1^ | |
| rs1851138 | 2 | 37 | 0.03 | 6.833 | 6.6×10^-2^ |  | 35 | 0.01 | 18.28 | 1.8×10^-5^ |  | 14 | 0.11 | 5.79 | 1.3×10^-1^ | |
| rs4235212 | 4 | 37 | 0.38 | 2.503 | 4.3×10^-2^ |  | 35 | 0.27 | 4.937 | 3.3×10^-5^ |  | 14 | 0.46 | 3.78 | 1.6×10^-1^ | |
| rs2241111 | 1 | 37 | 0.01 | 4.844 | 3.6×10^-1^ |  | 35 | 0.01 | 18.28 | 1.8×10^-5^ |  | 14 | 0.11 | 10.04 | 3.2×10^-3^ | |
| rs2758596 | 1 | 37 | 0.01 | 4.844 | 3.6×10^-1^ |  | 35 | 0.01 | 18.28 | 1.8×10^-5^ |  | 14 | 0.11 | 10.04 | 3.2×10^-3^ | |
| rs969348 | 15 | 37 | 0.09 | 5.866 | 4.7×10^-3^ |  | 35 | 0.10 | 5.907 | 1.8×10^-3^ |  | 14 | 0.43 | -4.13 | 9.3×10^-2^ | |
| rs10510382 | 3 | 37 | 0.23 | 3.033 | 1.2×10^-2^ |  | 35 | 0.30 | 3.842 | 2.6×10^-3^ |  | 14 | 0.14 | 7.72 | 1.9×10^-2^ | |
| rs17131753 | 1 | 37 | 0.03 | 7.784 | 3.5×10^-2^ |  | 35 | 0.01 | 18.28 | 1.8×10^-5^ |  | NA | NA | NA | 5.8×10^-4^ | |
| rs12511536 | 4 | 37 | 0.39 | 2.287 | 5.5×10^-2^ |  | 35 | 0.27 | 4.937 | 3.3×10^-5^ |  | 14 | 0.46 | 3.78 | 1.6×10^-1^ | |
| rs4425356 | 4 | 37 | 0.39 | 2.287 | 5.5×10^-2^ |  | 35 | 0.27 | 4.937 | 3.3×10^-5^ |  | 14 | 0.46 | 3.78 | 1.6×10^-1^ | |
| Abbreviations: CHR, chromosome; MAF, minor allele frequency; BETA: effect size for the minor allele in corresponding samples; Weight: sample size. NA indicates the SNP is monomorphic in the samples. | | | | | | | | | | | | | | | | |

| **Supplementary Table S1b**. Meta-analysis results of SNPs with *P* value less than 1 × 10^-5^ in the unadjusted GWAS of paclitaxel disposition. | | | | | | |
| --- | --- | --- | --- | --- | --- | --- |
|  |  | **Meta-analysis** | | | | |
| **SNP** | **CHR** | **Weight** | ***P*** | **Direction^a^** | **location** | **gene** |
| rs17130142 | 1 | 72 | 1.8×10^-7^ | ++? | intergenic | *LOC100505768 - PKN2* |
| rs1614627 | 1 | 44 | 6.0×10^-7^ | ?++ | intergenic | *CDA - PINK1* |
| rs11840576 | 13 | 72 | 1.5×10^-6^ | ++? | intergenic | *MYO16 - IRS2* |
| rs17211649 | 14 | 35 | 1.6×10^-6^ | ?-? | intronic | *RNASE4* |
| rs2056859 | 14 | 49 | 2.0×10^-6^ | ?-- | intergenic | *C14orf166B - IRF2BPL* |
| rs4899628 | 14 | 49 | 2.0×10^-6^ | ?-- | intergenic | *C14orf166B - IRF2BPL* |
| rs8083427 | 18 | 86 | 2.8×10^-6^ | +++ | intronic | *KCTD1* |
| rs9938946 | 16 | 86 | 3.4×10^-6^ | --- | intronic | *HS3ST4* |
| rs12134081 | 1 | 86 | 3.7×10^-6^ | --- | intergenic | *PTPRU - MATN1* |
| rs16838233 | 1 | 86 | 3.7×10^-6^ | +++ | intergenic | *PTPRU - MATN1* |
| rs16838255 | 1 | 86 | 3.7×10^-6^ | +++ | intergenic | *PTPRU - MATN1* |
| rs12425791 | 12 | 86 | 4.0×10^-6^ | +++ | intergenic | *NINJ2 - WNK1* |
| rs17169015 | 7 | 49 | 4.5×10^-6^ | ?++ | intronic | *PTN* |
| rs13331813 | 16 | 86 | 4.6×10^-6^ | --- | intronic | *HS3ST4* |
| rs12879016 | 14 | 86 | 5.1×10^-6^ | --+ | 3'UTR | *NRXN3* |
| rs2205226 | 14 | 86 | 5.1×10^-6^ | --+ | intergenic | *NRXN3 - DIO2* |
| rs1851138 | 2 | 86 | 5.5×10^-6^ | +++ | intergenic | *CCDC85A - VRK2* |
| rs4235212 | 4 | 86 | 5.7×10^-6^ | --- | intronic | *GLRA3* |
| rs2241111 | 1 | 86 | 6.0×10^-6^ | --- | upstream | *SLC25A44* |
| rs2758596 | 1 | 86 | 6.0×10^-6^ | +++ | intronic | *PMF1,PMF1-BGLAP* |
| rs969348 | 15 | 86 | 6.0×10^-6^ | +++ | intergenic | *TMC3 - MEX3B* |
| rs10510382 | 3 | 86 | 6.3×10^-6^ | --- | intergenic | *GRM7 - LOC100288428* |
| rs17131753 | 1 | 72 | 6.7×10^-6^ | ++? | intergenic | *NONE - LPHN2* |
| rs12511536 | 4 | 86 | 7.7×10^-6^ | +++ | intronic | *GLRA3* |
| rs4425356 | 4 | 86 | 7.7×10^-6^ | --- | intronic | *GLRA3* |
| Abbreviations: CHR, chromosome; BP, base pair; MAF, minor allele frequency; 3'UTR, three prime untranslated region; Weight: sample size.  ^a^The column ‘Direction’ indicates the effect direction of each variant, with respect to the minor allele in European samples, in the following subsets: Australian cohort (European ancestry), Dutch cohort (European ancestry) and Australian cohort (Asian ancestry) respectively. ‘?’ indicates the variant allele was not observed in the corresponding subset. ‘+’ indicates the variant allele was associated with increased paclitaxel T_C>0.05_ while ‘-’ indicates decreased paclitaxel T_C>0.05_. | | | | | | |

| **Supplementary Table S2a.** SNPs with meta-analysis *P* value less than 1 × 10^-5^ in the adjusted GWAS of paclitaxel disposition, stratified by cohorts. | | | | | | | | | | | | | | | |
| --- | --- | --- | --- | --- | --- | --- | --- | --- | --- | --- | --- | --- | --- | --- | --- |
|  |  | **Australian cohort European** | | | |  | **Dutch cohort European** | | | |  | **Australian cohort Asian** | | | |
| **SNP** | **CHR** | **Weight** | **MAF** | **BETA** | ***P*** |  | **Weight** | **MAF** | **BETA** | ***P*** |  | **Weight** | **MAF** | **BETA** | ***P*** |
| rs17130142 | 1 | 37 | 0.07 | 8.581 | 6.5×10^-6^ |  | 29 | 0.03 | 18.24 | 7.7×10^-5^ |  | NA | NA | NA | NA |
| rs9283636 | 3 | 37 | 0.43 | 3.049 | 1.9×10^-3^ |  | 29 | 0.47 | -4.52 | 6.7×10^-4^ |  | 14 | 0.32 | -3.97 | 3.5×10^-2^ |
| rs11840576 | 13 | 37 | 0.04 | 8.446 | 6.4×10^-4^ |  | 29 | 0.03 | 12.87 | 1.8×10^-4^ |  | NA | NA | NA | NA |
| rs1574560 | 3 | 37 | 0.43 | 3.049 | 1.9×10^-3^ |  | 28 | 0.47 | -4.53 | 8.6×10^-4^ |  | 14 | 0.32 | -3.97 | 3.5×10^-2^ |
| rs9787692 | 10 | 37 | 0.36 | 3.672 | 1.9×10^-3^ |  | 29 | 0.33 | 4.08 | 1.5×10^-3^ |  | 14 | 0.36 | 4.65 | 2.2×10^-2^ |
| rs7910940 | 10 | 37 | 0.34 | 3.668 | 3.3×10^-3^ |  | 29 | 0.30 | 4.40 | 3.4×10^-4^ |  | 14 | 0.36 | 3.46 | 9.0×10^-2^ |
| rs2483519 | 10 | 37 | 0.39 | 2.729 | 1.7×10^-2^ |  | 29 | 0.37 | 3.38 | 5.7×10^-3^ |  | 14 | 0.18 | 6.39 | 5.7×10^-4^ |
| rs7566539 | 2 | 37 | 0.05 | 7.171 | 9.2×10^-4^ |  | 29 | 0.06 | 8.14 | 7.7×10^-4^ |  | NA | NA | NA | NA |
| rs12140361 | 1 | 37 | 0.04 | 7.098 | 5.3×10^-3^ |  | 29 | 0.01 | 18.24 | 7.7×10^-5^ |  | NA | NA | NA | NA |
| rs7013285 | 8 | 37 | 0.07 | 3.067 | 7.4×10^-2^ |  | 29 | 0.07 | 12.30 | 7.8×10^-5^ |  | 14 | 0.04 | 40.72 | 8.6×10^-3^ |
| rs470903 | 18 | 37 | 0.19 | 5.453 | 1.1×10^-5^ |  | 29 | 0.13 | 4.00 | 5.8×10^-2^ |  | 14 | 0.18 | 4.36 | 2.0×10^-1^ |
| rs9934640 | 16 | 37 | 0.05 | 8.074 | 1.0×10^-4^ |  | 29 | 0.06 | 5.55 | 4.8×10^-2^ |  | 14 | 0.43 | 3.75 | 7.3×10^-2^ |
| rs2505127 | 10 | 37 | 0.16 | 5.005 | 4.4×10^-5^ |  | 29 | 0.11 | 4.68 | 1.8×10^-2^ |  | 14 | 0.43 | 1.82 | 4.3×10^-1^ |
| rs969348 | 15 | 37 | 0.09 | 6.056 | 4.0×10^-4^ |  | 29 | 0.10 | 6.00 | 4.1×10^-3^ |  | 14 | 0.43 | -2.60 | 3.5×10^-1^ |
| rs16969611 | 13 | 37 | 0.07 | 3.629 | 8.4×10^-2^ |  | 29 | 0.04 | 18.24 | 7.7×10^-5^ |  | 14 | 0.07 | 8.25 | 2.2×10^-2^ |
| rs9302891 | 17 | 37 | 0.05 | 6.616 | 3.3×10^-3^ |  | 29 | 0.04 | 18.24 | 7.7×10^-5^ |  | 14 | 0.07 | 1.03 | 8.0×10^-1^ |
| rs17741873 | 10 | 37 | 0.19 | 2.888 | 1.6×10^-2^ |  | 29 | 0.24 | 4.14 | 2.6×10^-3^ |  | 14 | 0.11 | 8.08 | 1.4×10^-2^ |
| rs595997 | 18 | 37 | 0.19 | 5.453 | 1.0×10^-5^ |  | 29 | 0.13 | 4.00 | 5.8×10^-2^ |  | 14 | 0.25 | 1.92 | 4.3×10^-1^ |
| rs305449 | 1 | 37 | 0.46 | 3.278 | 1.4×10^-4^ |  | 29 | 0.40 | 2.93 | 1.6×10^-2^ |  | 14 | 0.11 | -3.77 | 3.4×10^-1^ |
| Abbreviations: CHR, chromosome; BP, base pair; MAF, minor allele frequency; BETA: effect size for the minor allele in corresponding samples; Weight: sample size. NA indicates the SNP is monomorphic in the samples. | | | | | | | | | | | | | | | |

|  | **Supplementary Table S2b.** Meta-analysis results of SNPs with *P* value less than 1 × 10^-5^ in the adjusted GWAS of paclitaxel disposition. | | | | | | | | | | | | | | | | | | | | |  |
| --- | --- | --- | --- | --- | --- | --- | --- | --- | --- | --- | --- | --- | --- | --- | --- | --- | --- | --- | --- | --- | --- | --- |
|  |  | | |  | | **Meta-analysis** | | | | | | | | | | | | | | | |  |
|  | **SNP** | | | **CHR** | | **Weight** | | **P-value** | | **Direction^a^** | | | | **location** | | | **gene** | | | | |  |
|  | rs17130142 | | | 1 | | 66 | | 2.0×10^-9^ | | ++? | | | | intergenic | | | *LOC100505768 - PKN2* | | | | |  |
|  | rs9283636 | | | 3 | | 80 | | 4.5×10^-7^ | | +++ | | | | intergenic | | | *BCHE - ZBBX* | | | | |  |
|  | rs11840576 | | | 13 | | 66 | | 4.7×10^-7^ | | ++? | | | | intergenic | | | *MYO16 - IRS2* | | | | |  |
|  | rs1574560 | | | 3 | | 79 | | 5.8×10^-7^ | | --- | | | | intergenic | | | *BCHE - ZBBX* | | | | |  |
|  | rs9787692 | | | 10 | | 80 | | 6.6×10^-7^ | | --- | | | | intergenic | | | *GATA3 - NONE* | | | | |  |
|  | rs7910940 | | | 10 | | 80 | | 1.2×10^-6^ | | +++ | | | | intergenic | | | *GATA3 - NONE* | | | | |  |
|  | rs2483519 | | | 10 | | 80 | | 2.2×10^-6^ | | --- | | | | intronic | | | *ABLIM1* | | | | |  |
|  | rs7566539 | | | 2 | | 66 | | 2.5×10^-6^ | | ++? | | | | intergenic | | | *TSN - CNTNAP5* | | | | |  |
|  | rs12140361 | | | 1 | | 66 | | 2.5×10^-6^ | | --? | | | | intronic | | | *SDHC* | | | | |  |
|  | rs7013285 | | | 8 | | 80 | | 2.7×10^-6^ | | --- | | | | intronic | | | *OXR1* | | | | |  |
|  | rs470903 | | | 18 | | 80 | | 3.0×10^-6^ | | +++ | | | | intronic | | | *MBP* | | | | |  |
|  | rs9934640 | | | 16 | | 80 | | 4.8×10^-6^ | | --- | | | | intergenic | | | *WWOX - MAF* | | | | |  |
|  | rs2505127 | | | 10 | | 80 | | 5.8×10^-6^ | | +++ | | | | intergenic | | | *KIAA1462 - MTPAP* | | | | |  |
|  | rs969348 | | | 15 | | 80 | | 6.1×10^-6^ | | +++ | | | | intergenic | | | *TMC3 - MEX3B* | | | | |  |
|  | rs16969611 | | | 13 | | 80 | | 6.5×10^-6^ | | +++ | | | | intergenic | | | *ARGLU1 - FAM155A* | | | | |  |
|  | rs9302891 | | | 17 | | 80 | | 7.2×10^-6^ | | --- | | | | 5’UTR | | | *ABCA10* | | | | |  |
|  | rs17741873 | | | 10 | | 80 | | 7.5×10^-6^ | | +++ | | | | intergenic | | | *CAMK2G - C10orf55* | | | | |  |
|  | rs595997 | | | 18 | | 80 | | 7.9×10^-6^ | | --- | | | | intronic | | | *MBP* | | | | |  |
|  | rs305449 | | | 1 | | 80 | | 8.4×10^-6^ | | +++ | | | | intergenic | | | *LOC100505768 - PKN2* | | | | |  |
|  | Abbreviations: CHR, chromosome; BP, base pair; MAF, minor allele frequency; 5'UTR, five prime untranslated regions; Weight, sample size.  ^a^The column ‘Direction’ indicates the effect direction of each variant, with respect to the minor allele in European samples, in the following subsets: Australian cohort (European ancestry), Dutch cohort (European ancestry) and Australian cohort (Asian ancestry) respectively. ‘?’ indicates the variant allele was not observed in the corresponding subset. ‘+’ indicates the variant allele was associated with increased paclitaxel T_C>0.05_ while ‘-’ indicates decreased paclitaxel T_C>0.05_. | | | | | | | | | | | | | | | | | | | | |  |
| **Supplementary Table S3.** Results of SNPs with *P* value less than 1 × 10^-5^ in the unadjusted GWAS of carboplatin clearance. | | | | | | | | | | | | | | | | | | | | | | |
|  | |  |  | | **European (n = 42)** | | | | | |  | **Asian (n = 14)** | | | |  | | **Meta-analysis (n = 56)** | | | | |
| **SNP** | | **CHR** | **BP** | | **MAF** | | **BETA** | | ***P*** | |  | **MAF** | **BETA** | | ***P*** |  | | ***P*** | **Direction***^a^* | **Location** | **Gene** | |
| rs12516561 | | 5 | 137445000 | | 0.14 | | 1.671 | | 6.9 × 10^-4^ | |  | 0.36 | 2.153 | | 8.1 × 10^-4^ |  | | 3.9 × 10^-6^ | ++ | intergenic | *WNT8A* and *NME5* | |
| rs9558996 | | 13 | 107942432 | | 0.23 | | 1.285 | | 1.2 × 10^-4^ | |  | 0.04 | 5.1 | | 3.7 × 10^-4^ |  | | 4.7 × 10^-6^ | ++ | intronic | *FAM155A* | |
| rs17806780 | | 4 | 146732654 | | 0.17 | | 1.484 | | 2.1 × 10^-4^ | |  | 0.04 | 5.1 | | 3.7 × 10^-4^ |  | | 8.6 × 10^-6^ | -- | intronic | *ZNF827* | |
| rs11130471 | | 3 | 55411011 | | 0.19 | | -1.641 | | 9.3 × 10^-5^ | |  | 0.29 | 1.253 | | 3.4× 10^-2^ |  | | 8.9 × 10^-6^ | -- | intergenic | *CACNA2D3* and *WNT5A* | |

Abbreviations: SNP, single nucleotide polymorphism; CHR, chromosome; BP, base pairs; MAF, minor allele frequency; BETA: effect size for the minor allele in corresponding samples.

^a^The column ‘Direction’ indicates the effect direction of each variant, with respect to the minor allele in European samples, in the Australian cohort of European ancestry and Asian ancestry respectively, ‘+’ indicates the variant allele was associated with increased carboplatin clearance while ‘-’ indicated decreased clearance.

| **Supplementary Table S4.** Results of SNPs with *P* value less than 1 × 10^-5^ in the adjusted meta-analysis of carboplatin clearance. | | | | | | | | | | | | | | | |
| --- | --- | --- | --- | --- | --- | --- | --- | --- | --- | --- | --- | --- | --- | --- | --- |
|  |  |  | **European (n = 42)** | | |  | **Asian ( n = 14)** | | |  | **Meta-analysis** | | | | |
| **SNP** | **CHR** | **BP** | **MAF** | **BETA** | ***P*** |  | **MAF** | **BETA** | ***P*** |  | **Weight** | ***P*** | **Direction**^a^ | **Location** | **Gene** |
| rs7230264 | 18 | 24927434 | 0.12 | -0.65 | 1.0 × 10^-4^ |  | 0.25 | -0.65 | 1.9 × 10^-3^ |  | 56 | 8.7 × 10^-7^ | -- | intronic | *RP11-739N10* |
| rs12412836 | 10 | 71669953 | 0.10 | -0.75 | 3.5 × 10^-5^ |  | 0.2143 | -0.50 | 3.9 × 10^-2^ |  | 56 | 3.9 × 10^-6^ | -- | intronic | *COL13A1* |
| rs12165173 | 2 | 217875139 | 0.05 | -1.06 | 4.9 × 10^-6^ |  | NA | NA | NA |  | 42 | 4.9 × 10^-6^ | + | intergenic | *TNP1* - *DIRC3* |
| AC | 10 | 101595996 | 0.04 | -1.21 | 5.2 × 10^-6^ |  | NA | NA | NA |  | 42 | 5.2 × 10^-6^ | - | exonic | *ABCC2* |
| rs8187707 | 10 | 101610533 | 0.04 | -1.21 | 5.2 × 10^-6^ |  | NA | NA | NA |  | 42 | 5.2 × 10^-6^ | - | exonic | *ABCC2* |
| rs8187710 | 10 | 101611294 | 0.04 | -1.21 | 5.2 × 10^-6^ |  | NA | NA | NA |  | 42 | 5.2 × 10^-6^ | - | exonic | *ABCC2* |
| rs11816708 | 10 | 101615015 | 0.04 | -1.21 | 5.2 × 10^-6^ |  | NA | NA | NA |  | 42 | 5.2 × 10^-6^ | - | intergenic | *ABCC2* - *DNMBP* |
| rs17280534 | 14 | 97244455 | 0.02 | -1.49 | 6.4 × 10^-6^ |  | NA | NA | NA |  | 42 | 6.4 × 10^-6^ | - | intergenic | *PAPOLA* - *VRK1* |
| rs17635967 | 7 | 151184920 | 0.02 | -1.49 | 6.4 × 10^-6^ |  | NA | NA | NA |  | 42 | 6.4 × 10^-6^ | + | intronic | *RHEB* |
| rs4313770 | 15 | 38782493 | 0.02 | -1.49 | 6.4 × 10^-6^ |  | NA | NA | NA |  | 42 | 6.4 × 10^-6^ | - | UTR3 | *RASGRP1* |
| rs7102974 | 11 | 61560035 | 0.02 | -1.49 | 6.4 × 10^-6^ |  | NA | NA | NA |  | 42 | 6.4 × 10^-6^ | - | UTR5 | *C11orf10* |
| rs7909167 | 10 | 25891244 | 0.02 | -1.49 | 6.4 × 10^-6^ |  | NA | NA | NA |  | 42 | 6.4 × 10^-6^ | - | downstream | *GPR158* |
| rs4692174 | 4 | 27186432 | 0.07 | -0.88 | 6.6 × 10^-6^ |  | NA | NA | NA |  | 42 | 6.6 × 10^-6^ | + | intergenic | *STIM2* - *MIR4275* |
| rs12571637 | 10 | 86801185 | 0.01 | -1.59 | 9.0 × 10^-4^ |  | 0.07143 | -1.29 | 1.1 × 10^-3^ |  | 56 | 6.8 × 10^-6^ | ++ | intergenic | *FAM190B* - *LOC100507470* |
| rs2202766 | 6 | 106237505 | 0.02 | -1.47 | 7.4 × 10^-6^ |  | NA | NA | NA |  | 41 | 7.4 × 10^-6^ | - | intergenic | *PREP - PRDM1* |
| rs9982086 | 21 | 27004109 | 0.05 | -1.06 | 8.8 × 10^-6^ |  | NA | NA | NA |  | 42 | 8.8 × 10^-6^ | + | intergenic | *MRPL39* - *JAM2* |
| rs5741804 | 20 | 36952342 | 0.02 | -1.49 | 6.4 × 10^-6^ |  | 0.3214 | -0.23 | 2.9 × 10^-2^ |  | 56 | 9.2 × 10^-6^ | -- | exonic | *BPI* |

Abbreviations: SNP, single nucleotide polymorphism; CHR, chromosome; BP, base pairs; MAF, minor allele frequency; BETA: effect size for the minor allele in corresponding samples: Weight: sample size. NA indicates the SNP is monomorphic in the samples.

^a^ The column ‘Direction’ indicates the effect direction of each variant, with respect to the minor allele in European samples, in the Australian cohort of European ancestry and Asian ancestry respectively, ‘+’ indicates the variant allele was associated with increased carboplatin clearance while ‘-’ indicated decreased clearance.
